# Hydrating Capabilities of the Biopolymers Produced by the Marine Thermophilic *Bacillus horneckiae* SBP3 as Evaluated by ATR-FTIR Spectroscopy

**DOI:** 10.3390/ma15175988

**Published:** 2022-08-30

**Authors:** Maria Teresa Caccamo, Vincenzo Zammuto, Antonio Spanò, Concetta Gugliandolo, Salvatore Magazù

**Affiliations:** 1 Department of Mathematical and Computer Sciences, Physical Sciences and Earth Sciences, University of Messina, Viale Ferdinando Stagno D’Alcontres 31, 98166 Messina, Italy; 2 Department of Chemical, Biological, Pharmaceutical and Environmental Sciences, University of Messina, Viale Ferdinando Stagno D’Alcontres 31, 98166 Messina, Italy; 3 Research Centre for Extreme Environments and Extremophiles, University of Messina, Viale Ferdinando Stagno D’Alcontres 31, 98166 Messina, Italy

**Keywords:** biosurfactant, infrared spectroscopy, *Bacillus*, extremophiles, wetting agents

## Abstract

The surfactin-like lipopeptide (BS-SBP3) and the exopolysaccharide (EPS-SBP3) produced by the polyextremophilic *Bacillus horneckiae* SBP3 (DSM 103063) have been recently described as valuable biopolymers useful in biotechnological applications. To investigate the hydrating capabilities of BS-SBP3 and EPS-SBP3, here we evaluated (i) their wetting properties, measuring the contact angle; (ii) their moisture uptake abilities using the gravimetric method; and (iii) their hydrating states (from 0 to 160% *w*/*w* of water content) using ATR-FTIR spectroscopy. BS-SBP3 reduced the water contact angle on a hydrophobic surface from 81.7° to 51.3°, whereas the contact angle in the presence of EPS-SBP3 was 72.9°, indicating that BS-SBP3 improved the wettability of the hydrophobic surface. In the moisture uptake tests, EPS-SBP3 absorbed more water than BS-SBP3, increasing its weight from 10 mg to 30.1 mg after 36 h of 100% humidity exposure. Spectral distance and cross-correlation analyses were used to evaluate the molecular changes of the two biopolymers during the hydration process. As the water concentration increased, BS-SBP3 spectra changed in intensity in the two contributions of the OH-stretching band named “closed” and “open” (3247 and 3336 cm^−1^, respectively). Differently, the spectra of EPS-SBP3 exhibited a broader peak (3257 cm^−1^), which shifted at higher water concentrations. As evaluated by the spectral distance and the wavelet cross-correlation analysis, the OH-stretching bands of the BS-SBP3 and EPS-SBP3 changed as a function of water content, with two different sigmoidal trends having the inflection points at 80% and 48%, respectively, indicating peculiar water-properties of each biopolymer. As wetting agents, these biopolymers might replace industrially manufactured additives in agriculture and the food and cosmetic industries.

## 1. Introduction

Since the global water reserves have been significantly depleted by past exploitation, water will be the most valuable resource for agriculture in the future. In addition, climate change predictions indicate that precipitation-supplied water for agriculture is decreasing, and several countries are struggling with an ever-increasing rate of desertification and water scarcity [1]. The potential implications for humankind are cataclysmic, as decreased food production on vulnerable soils will increase stress on a growing global population [2,3]. The presence of scarce quantities of water in arid or semi-arid soils can form a water-repellent barrier that limits the rate and capacity of water absorption. This phenomenon is called water repellency [4]. In some arid areas, water repellency makes it impossible to farm without expensive improvements [5]. There is a great deal of interest in the development of strategies to combat water repellency; among the most used are the addition of clays to increase the surface of the soil particles, the working of the soil to crush and abrade hydrophobic surfaces, and the use of wetting agents of chemical synthesis such as cement and polyacrylamide [6,7]. The phenomenon of water repellency is increasing all over the world, but its control with wetting agents has been shown to boost crop yields and lessen the impact of plant diseases [8]. Due to the low environmental compatibility of the traditional wetting agents, multimillion-dollar companies have dedicated themselves to the search for new compounds able to absorb water and reduce water repellency [9]. Recently, the incorporation of biopolymers, which are environmentally friendly and eco-compatible, in soils is proposed as one technique to reduce soil water repellency. The addition of biopolymers with polysaccharidic structures (such as xanthan gum or gellan gum) at a concentration of 2% by weight increases the water permeability of sand of various gradations by two to four orders of magnitude [10,11,12]. In addition to sandier soils, the use of 1% xanthan gum successfully lowered the saturated water permeability of clayey soils by more than 80% [13]. While the polysaccharidic compounds can counter water repellency, absorbing water due to the presence of various functional groups, including hydroxyl groups, the surface-active biopolymers or biosurfactants (BSs) are known to be able to modify the properties of soil particles, including their wettability.

Bacteria are known to produce a wide range of exopolymers, such as BSs and exopolysaccharides (EPSs), that are essential for increasing the bioavailability of nutrients or forming architecturally complex biostructures called biofilms [14,15,16].

EPSs produced by bacteria represent a strategy for growth and survival in adverse conditions. These biopolymers are involved in different cell functions such as adhesion to abiotic and biotic surfaces, protection of cells, modulation of cell-to-cell signaling, and cell environmental sensing [5]. EPSs are also the major constituents of the biofilm matrix, in which bacterial cells are protected against environmental stress conditions (high or low temperature, salinities, nutrient availability, antibiotics), and they serve as a water and nutrient reservoir [16,17]. The role of EPSs in affecting the hydrological behavior of the natural biological crust has been a matter of study over the years. EPS-producing cyanobacteria were used to promote the stabilization of desert soils [18,19,20]. EPSs have been reported to reduce the ease of water penetration into the soil by inducing surface sealing and clogging of pores, thus affecting hydraulic conductivity [21,22] and allowing the creation of moistened microenvironments that retain water for longer periods than the surroundings [23]. Furthermore, it was proved that they are essential for water adsorption to soils with low hydraulic conductivity [24]. Notwithstanding the importance attributed to EPSs, few studies are present on their hydration properties at the molecular level to clarify the mechanisms of the hydration processes of these biopolymers.

BSs have been the subject of increasing interest in recent years; a wide range of microorganisms synthesize them. These biopolymers possess an amphipathic nature with diverse chemical structures, including glycolipids, lipopeptides, polysaccharide–protein complexes, phospholipids, fatty acids, and neutral lipids [14,15]. Due to their surface activity, BSs could be applied in fields related to emulsification, foaming, detergency, wetting, dispersion, and solubilization of hydrophobic compounds [15]. The advantages of BSs consist of their lower toxicity; higher biodegradability; and effectiveness at extreme temperatures, salinities, and pH values that make them a green alternative to their chemical counterparts in different applications, including agriculture, food, cosmetics, and petroleum industries, as well as in bioremediation [14,15,25,26]. Several marine microorganisms able to produce biosurfactants with different structures have been previously reported by Gudiña et al. [15].

In the Mediterranean Sea, the shallow hydrothermal vent of the Eolian Islands (Italy) represents an extreme marine environment characterized by unusual conditions for most organisms (high temperature, high concentrations of H_2_S, CO_2_, etc.) [27]. Eolian shallow vents are a source of thermophilic and thermotolerant *Bacillus* strains able to produce novel EPSs and BSs potentially valuable for responding to the increasing demand for new active bioproducts for biotechnological purposes [28,29,30,31,32,33,34,35].

Several EPSs from Eolian thermophilic bacilli, such as those from *B. licheniformis* strains B3-15 [28] and T14 [29] and *G. thermodenitrificans* B3-72 [30], have been reported to possess unique properties, mainly thermostability and non-cytotoxicity, and biological activities, including antibiofilm, antiviral, and immunostimulant effects, that make them potentially useful in different biotechnological applications [27,28,29,30,31,32,33,34].

Bacilli able to utilize hydrocarbons were previously isolated from different Eolian vents, including the thermophilic *Geobacillus* strains (5-2, 10-1, and 1 bw) [31]. Since the ability to grow in the presence of hydrocarbons is related to the production of BSs, it is reasonable to suppose that bacilli present in shallow hydrothermal vents can produce BSs. Recently, the thermophilic *Bacillus horneckiae* SBP3 and *B. licheniformis* B3-15, isolated from shallow hydrothermal vents of the Eolian Island (Italy), have been reported as producers of different surfactin- and lichenysin-like lipopeptides with surfactant activity that can be considered of high value in biotechnological applications [35]. Furthermore, their CFSs demonstrated significant potential for emulsifying both hydrocarbons and vegetable oils, implying a potential use in bioremediation and cleaning petrol-contaminated surfaces such as oil pipelines, bilge tankers, and industrial silos, as well as in cleaning solutions in the food industry [35].

The EPSs and BSs produced by these bacilli have not previously been studied for their hydrating capabilities. This type of study could lead to new applications that include moisturizing agents or novel wetting agents used to improve the quality of soils.

Although our previous studies have characterized the thermal behavior at the molecular level of bacterial EPSs by spectroscopic techniques [36,37], to our knowledge, no spectroscopic studies have been performed on the hydrating process of EPSs and BSs produced by marine thermophilic strains.

To gain structural information on the investigated hydrated biological polymers, attenuated total reflectance Fourier-transform infrared (ATR-FTIR) spectroscopy was reported as a powerful technique since it enables overcoming some analytical limitations (e.g., in the dry compounds or during the first hydration events) with respect to other well-established techniques. This spectroscopic technique allowed us to study (i) the changes of the spectral bands associated with distinct functional macromolecule groups as a function of water content and (ii) the variation of water structure molecules [38,39,40]. H_2_O displays a strong contribution to the ATR-FTIR spectrum of macromolecules and changes during the different hydration conditions [41,42,43]. The spectral changes observed as a function of water content may be monitored, correlated to the changes in macromolecule conformation, and used to identify the sites of water sorption. The OH-stretching band, coupled with an appropriate mathematical approach, can be used to describe the hydration processes [44,45,46]. The ATR-FTIR technique has been applied to describe the interaction of water with different macromolecules such as melanin, collagen, lysozyme, and lipids [40,47].

In order to investigate hydrating capabilities of the surfactant (BS-SBP3) and the exopolysaccharide (EPS-SBP3) produced by *B. horneckiae* SBP3, in this study we evaluated (i) the wetting properties, measuring the contact angle of water solution on a hydrophobic surface; (ii) the moisture uptake, using the gravimetric method; and (iii) the hydrating states (from 0 to 160% *w*/*w* of water content) using ATR-FTIR spectroscopy. To extract quantitative information on the behavior of the water content in the two biopolymers, two mathematical tools for each spectrum are used: (i) spectral distance and (ii) wavelet cross-correlation analysis [36,37]. Information on the hydration state of the biopolymers could allow us to expand their potential use for biotechnology and agriculture as wetting agents to counteract the desertification of soils.

## 2. Materials and Methods

### 2.1. Bacterial Strain

*Bacillus horneckiae* SBP3 was isolated from a sample in June 2006 close to the shallow hydrothermal vent near the island of Panarea (Eolian Islands, Italy), named Black Point (coordinates: 38° 38′023” N–50°60′28” E, depth: 23 m) The properties of the fluid emitted from Black Point are the following: temperature equal to 130 °C, pH value equal to 3.0, and conductivity equal to 46.20 mS/cm [27]. The strain was routinely grown on Marine Agar 2216 (MA, Difco Laboratories, Detroit, MI, USA) and frozen at −80 °C in 40% (*v*/*v*) glycerol for long-term storage.

### 2.2. Production and Characterization of the Biopolymers from SBP3

To produce the biosurfactant (BS-SBP3), the SBP3 strain was grown aerobically in marine broth (Oxoid) supplemented with 3% sucrose (MB+SAC) and incubated at 45 °C in a rotary shaker at 130 rpm for 48 h as previously reported [35]. The acid precipitation method was used to extract the biopolymer from the cell-free supernatant (CFS). The CFS was acidified utilizing 2 N HCl to reach the 2.0 pH value and kept overnight to form precipitation. The biosurfactant was separated using 2:1 (*v*/*v*) chloroform and methanol, and the organic layer was separated to collect the concentrated biosurfactant using a rotary evaporation process and lyophilized [35].

The exopolysaccharide (EPS-SBP3) was produced as reported by Gugliandolo et al., [27]. Briefly, the strain was cultivated in a minimal medium containing 0.01% yeast extract and seawater supplemented with 1% *w/v* of sucrose (SWY+SAC). The supernatant was treated with an equal volume of absolute cold ethanol, added dropwise under stirring in an ice bath, held at −20 °C overnight, and then centrifuged at 13,000× *g* for 30 min. The pellet was washed twice with ethanol, dissolved in hot water, dialyzed against distilled water, lyophilized, and weighed.

The dried BS-SBP3 and EPS-SBP3 were analyzed in triplicate by attenuated total reflectance Fourier-transform infrared (ATR-FTIR) spectroscopy. An ATR-FTIR Vertex 70 V spectrometer (Bruker Optics, Germany) using platinum diamond ATR was employed to collect spectra in the 4000 to 500 cm^−1^ wavenumber range. The analysis of IR spectra was carried out by using the Origin Lab Co software (Origin Lab Co., Northampton, MA, USA).

### 2.3. CFS Surface Tension and Emulsifying Properties

Three replicates of each culture were centrifuged at 3800× *g* for 20 min at 4 °C to produce the CFSs.

The reduction in surface tension was measured (in triplicate) using a digital Wilhelmy plate-type tensiometer K10T (Krǖss, Hamburg, Germany) [48].

The emulsifying activity of CFSs from aerobically cultivated strains in SWY+SAC and MB+SAC was assessed according to Cooper and Goldenberg [49], with a few adjustments. In a glass tube (10 cm in height and 1 cm in diameter), an aliquot of each CFS (2 mL) was combined with an equal amount of kerosene and swirled vigorously in the vortex for two minutes. The uncultured media served as a negative control. After mixing with kerosene for 24 h, the emulsifying index (E_24_) was computed as the difference between the height of the emulsion layer and the total height, multiplied by 100.

### 2.4. Wetting Property

To characterize the wetting properties of hydrophobic surfaces modified by different biopolymer solutions (BS-SBP3 and EPS-SBP3) at increasing concentrations ranging from 0 to 10 mg/mL, the contact angle (θ) was determined using the sessile drop technique on a hydrophobic substrate (polystyrene) at 25 °C. The image was captured with a high-resolution camera and analyzed using the software ImageJ DropSnake plugin to measure θ on sessile drop images as reported by Daerr [50]. The measurements were repeated thrice, and the average value is reported.

### 2.5. Moisture Uptake

The moisture uptake was evaluated as described previously by Raddadi et al. [51]. The dried biopolymers (10 mg) were incubated in Petri dishes in a sealed desiccator. To ensure high relative humidity, the bottom of the desiccator was filled with sterile tap water and was incubated for 36 h at a constant temperature (30 °C). The samples were weighed to evaluate their weight increase due to humidity uptake.

### 2.6. Hydrating States of BS-SBP3 and EPS-SBP3 Using ATR-FTIR Spectroscopy

To dynamically determine the hydrating states of biopolymers, before ATR-FTIR analysis, the following quantities of double-distilled water were added to the lyophilized BS-SBP3 or EPS-SBP3: 0, 20, 40, 60, 80, 100, 120, 140, and 160% *w*/*w* [52].

ATR-FTIR spectroscopy allows obtaining qualitative information about the anhydrous BS-SBP3 or EPS-SBP3 and the two hydrated biopolymers. To collect the spectra of anhydrous and progressively hydrated BS-SBP3 or EPS-SBP3, an ATR-FTIR Vertex 70 V spectrometer from Bruker Optics with a platinum diamond was used. The ATR-FTIR spectra were collected using an average of 64 scans with a resolution of 4 cm^−1^ in the spectral range of 500 ÷ 4000 cm^−1^. To decrease the instrumental noise of the spectra, the data were pre-processed using Bruker OPUS software, performing (i) the baseline and (ii) the smoothing treatments. Finally, a procedure to normalize the spectra, with specific reference to some bands having the same intensity in each region, has allowed reducing the differences between one measurement and another [53]. After this procedure, data were processed in a MATLAB environment.

### 2.7. Spectral Distance

The spectral distance (SD) gives the deviation of the anhydrous spectrum at water content ranging from anhydrous to 160% water added in the two biopolymers (BS-SBP3 and EPS-SBP3). For this purpose, in this work, we calculated the SD of the spectra, employing the following expression:

(1) $$SD=\sqrt{\underset{v}{\sum }{\left[I\left(\omega ,s\right)-I\left(\omega ,s_{r}\right)\right]}^{2}\Delta \omega }$$

In our study, I(ω) is the absorbance at frequency ω, s represents the concentration of water, $s_{r}$ refers to the anhydrous biopolymer, and Δω is the frequency resolution. Due to the high sensitivity of the employed technique to water, the SD variations are mainly determined by the structural rearrangements of the H-bond network [39]. To quantitatively extract information about the OH-stretching band, a model fit for the SD versus the water content was adopted:

(2) $$SD=A-\frac{A}{1+e^{-B\left(c-c_{0}\right)}}+\left(C-Dc\right)$$

where A is the sigmoid amplitude, *B* represents the sigmoid steepness, $c_{0}$ indicates the value of the sigmoid inflection point, and *C − Dc* refers to a linear base contribution [36,37].

### 2.8. Cross-Correlation Wavelet

As a rule, the cross-correlation wavelet (XWT) gives the degree of affinity between two spectral signals [36,54,55]; in our case, evaluating it in whole the concentration range allows us to estimate the behavior trend when a reference spectrum is considered the spectrum of the anhydrous biopolymer. The wavelet cross-correlation coefficients (XWTCs) have been calculated as follows:

(3) $$XWT_{C}=\frac{\int^{\;}W_{1}\left(a,\tau \right)W_{2}^{*}\left(a,\tau \right)d\tau }{\sqrt{WS_{1}\left(a\right)WS_{2}\left(a\right)}}$$

where $W_{1}$ and $W_{2}$ represent the wavelet transform and * is the complex conjugation, a is the scale parameter (a>0), τ is the parameter of shift, and $WS_{1}$ and $WS_{2}$ are wavelet spectra.

## 3. Results

### 3.1. CFS Properties of B. horneckiae SBP3

The surface tension (ST) and the ability to emulsify kerosene of each CFS are reported in Table 1.

The highest ST reduction and E_24_ value on kerosene were obtained from the CFS of SBP3 grown in MB+SAC, while the CFS from SWY+SAC was only able to form a stable emulsion with kerosene.

We extracted EPS-SBP3 from SWY+SAC as reported by Gugliandolo et al. [27], and its yield was 70 mg L^−1^. Conversely, the BS-SBP3 was extracted from MB+SAC, and the yield was 950 mg L^−1^ after 48 h [35].

### 3.2. The FTIR Analysis of the Biopolymers

**Figure 1:**
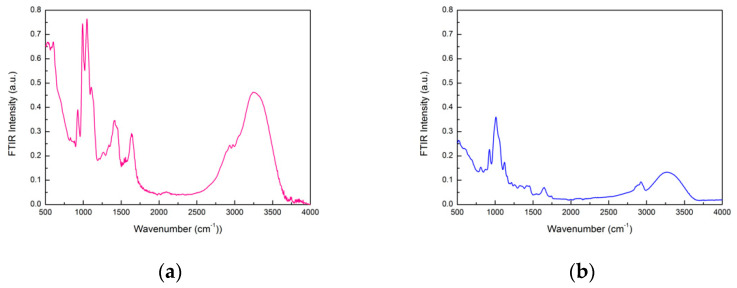
(**a**) ATR-FTIR spectrum of BS-SBP3 in the spectral range 500–4000 cm^−1^; (**b**) ATR-FTIR spectrum of EPS-SBP3 in the spectral range 500–4000 cm^−1^.

The spectra obtained using the ATR-FTIR technique on BS-SBP3 and EPS-SBP3 in the spectral range 500–4000 cm^−1^ are reported in Figure 1a,b, respectively.

As far as the peak wavenumber assignment is concerned (see Table 2), the spectrum of BS-SBP3 shows a broad band contribution which can be assigned to the intramolecular OH-stretching contribution in the region between ~3700 cm^−1^ and ~3000 cm^−1^. The peaks at ~2979 cm^−1^ and ~2938 cm^−1^ are assigned to C-H stretching, while the peak centered at 1645 cm^−1^ can be assigned to the H-O-H-bending contribution. Therefore, a lipopeptide structure like surfactin was attributed to the BS-SBP3. Figure 1b shows the EPS-SBP3 spectrum with the following features: the broad stretching contribution in the 3500–3000 cm^−1^, 1200–950 cm^−1^, and 970–920 cm^−1^ frequency regions can be related to the vibrations of carbohydrate hydroxyl groups considered as the fingerprint regions of exopolysaccharides [36,37,56,57].

The intramolecular OH-stretching (3500–3000 cm^−1^) contribution in water and aqueous solutions is usually analyzed following different interpretative models that refer to different arrangements of water molecules.

### 3.3. Wettability Assay

**Figure 2:**
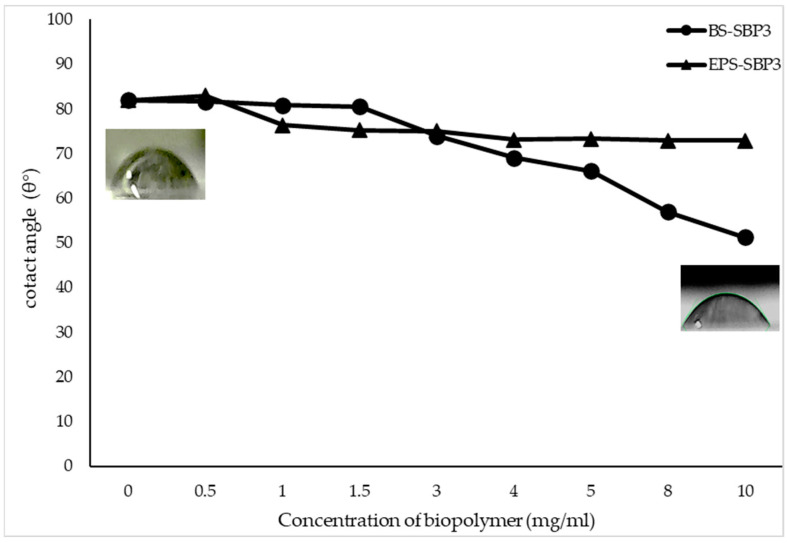
Contact angle of the biopolymers BS-SBP3 and EPS-SBP3 at an increasing concentration from 0 to 10 mg/mL.

The contact angles of BS-SBP3 and EPS-SBP3 on the hydrophobic substrates were 51.3° and 72.9°, respectively. This showed that BS-SBP3 had a better wetting performance than EPS-SBP3, and it spread more easily on the surface (Figure 2).

A contact angle of less than 90° usually implies a favorable wetting of surfaces, hence easily spreading wetting over a large area of solid surfaces. Conversely, contact angles above 90° generally indicate that wetting of the surface is unfavorable, so the fluids may minimize contact with the surface and form compact droplets.

### 3.4. Moisture Uptake Assay

**Figure 3:**
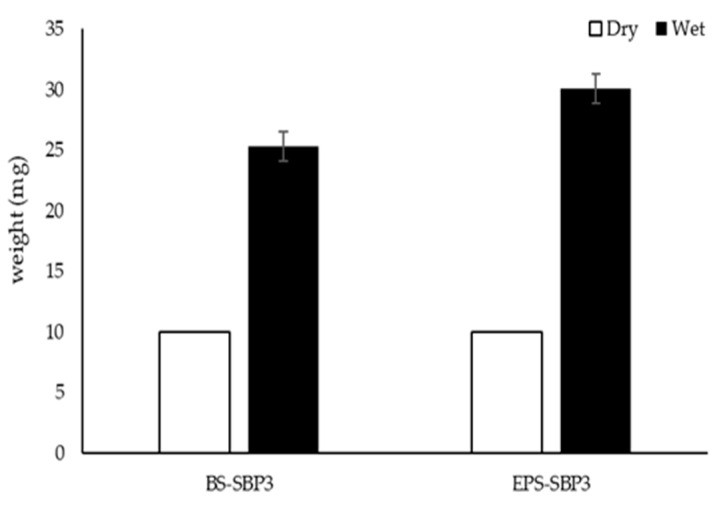
Weight of the BS-SBP3 and EPS-SBP3 before (dry) after (wet) 36 h of exposure to 100% air humidity.

As shown in Figure 3, the biopolymers produced by SBP3 were able to increase their weight due to the large quantity of water absorbed.

Data reported in Figure 3 show the increase in weight of EPS-SBP3 from 10 mg to 30.1 mg, indicating a greater moisture uptake than BS-SBP3 (25.3 mg).

### 3.5. The Hydration State of Biopolymers by ATR-FTIR Analysis

**Figure 4:**
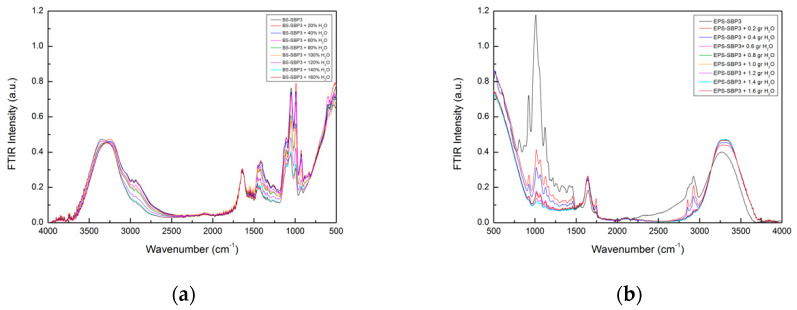
Infrared spectra for BS-SBP3 aqueous solutions (**a**) and EPS-SBP3 aqueous solutions (**b**) at different water concentrations in the spectral range 500–4000 cm^−1^.

The ATR-FTIR spectra in the 4000–500 cm^−1^ spectral range of BS-SBP3 and EPS-SBP3 at increasing water content (i.e., 0, 20, 40, 60, 80, 100, 120, 140, and 160%) are shown in Figure 4. The experimentally registered spectra have been normalized for the background. As far as the high-frequency region is concerned (i.e., for Δω > 2400 cm^−1^), the shape of the intramolecular OH-stretching band shows significant changes with concentration. Furthermore, as it can be seen, a shift in OH stretching with increasing water content is present.

**Figure 5:**
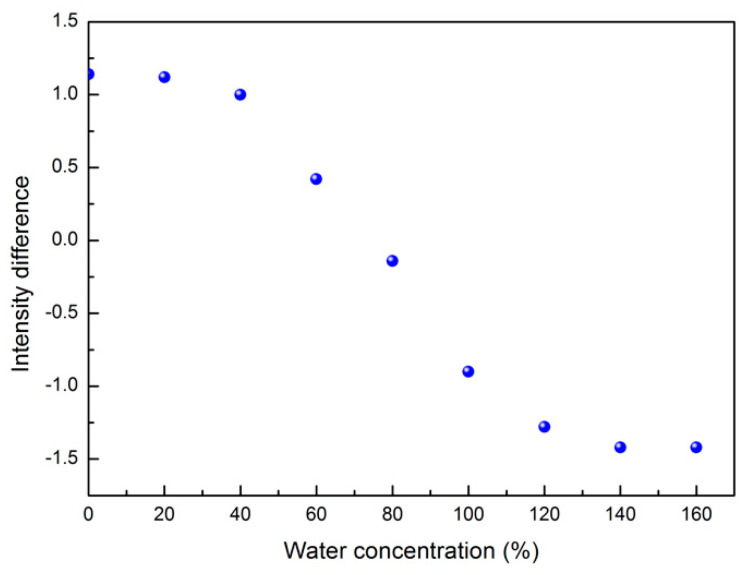
Peak intensity difference between the contribution centered at ~3247 cm^−1^ and the contribution centered at ~3330 cm^−1^ as a function of water concentration for BS-SBP3.

The OH-stretching band of the BS-SBP3 spectra shows two main sub-bands centered at ~3247 cm^−1^ and ~3336 cm^−1^, respectively, separated by the presence of an isosbestic point. On the contrary, the OH-stretching band of EPS-SBP3 shows non-clearly distinguished sub-bands. However, when the water content is increased, the peak at ~3257 cm^−1^ shifts to higher wavenumber values. To extract information from the OH-stretching band, the peak intensity difference at ~3247 cm^−1^ and ~3336 cm^−1^ as a function of water concentration has been evaluated, and the obtained data are reported in Figure 5.

As it can be seen, the intensity difference data as a function of the water content fulfill a decreasing sigmoidal trend characterized by distinguished linear regions separated by two kinks localized at water concentration values of 40% and 120%.

**Figure 6:**
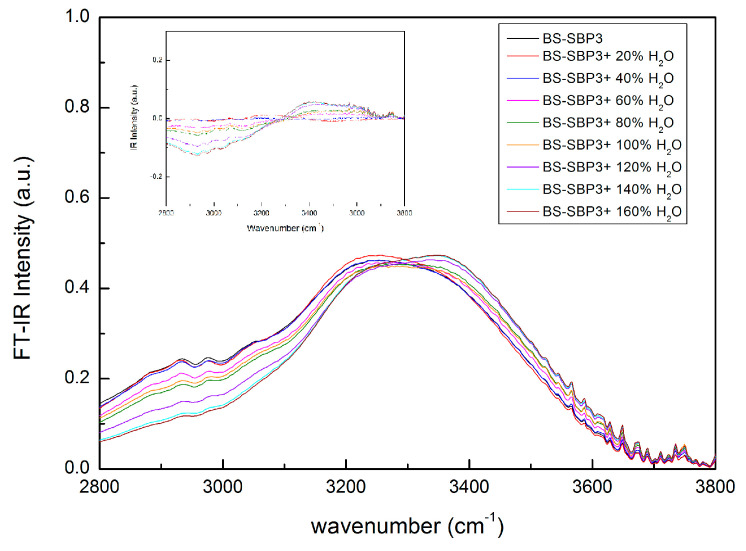
Infrared spectra of the intramolecular O-H-stretching bands collected for BS-SBP3 aqueous solutions at different water contents. In the inset, the difference between the hydrated BS-SBP3 spectra and the anhydrous BS-SBP3 spectrum at different concentration values is reported.

It is well known that in the “two-state” water model, the water is conceived as a mixture of two different configurations with different intermolecular bonding coordination [60,61,62]. The O-H-stretching band of BS-SBP3 spectra at different states of hydration is shown in Figure 6, which clearly shows the shift of the peaks of the intramolecular OH-stretching bands. In particular, the shape of this contribution is almost the same for the anhydrous BS-SBP3 (black) and the BS-SBP3 with 0.2 g of water (red) samples; starting from the concentration of 0.4 g of water content (blue curve), the whole band shows a decrease in its low-frequency contribution (~3247 cm^−1^), while the high-frequency contribution (~3330 cm^−1^) increases.

Furthermore, a noteworthy feature of the registered spectra is that starting from anhydrous BS-SBP3, after an increase in water content, there is a restricted spectral region in which the IR absorption intensity maintains an almost constant value; in other terms, the presence of an isosbestic point is registered. The presence of this almost constant intensity point which does not change when an intensive parameter, i.e., concentration, changes implies that some of the integrated intensity flows from one side to the other one of the total spectrum, indicating an equilibrium between the free and bonded water contributions. To better show this OH-stretching mode’s peculiar behavior, the inset of Figure 6 reports the difference between the BS-SBP3 aqueous solution spectra and the anhydrous BS-SBP3 spectrum.

As it can be seen in the inset of Figure 6, the difference spectra as a function of water concentration show a marked intensity shift which reveals a change in the water population from a dominant closed configuration to a dominant open configuration.

### 3.6. Spectral Distance Analysis

**Figure 7:**
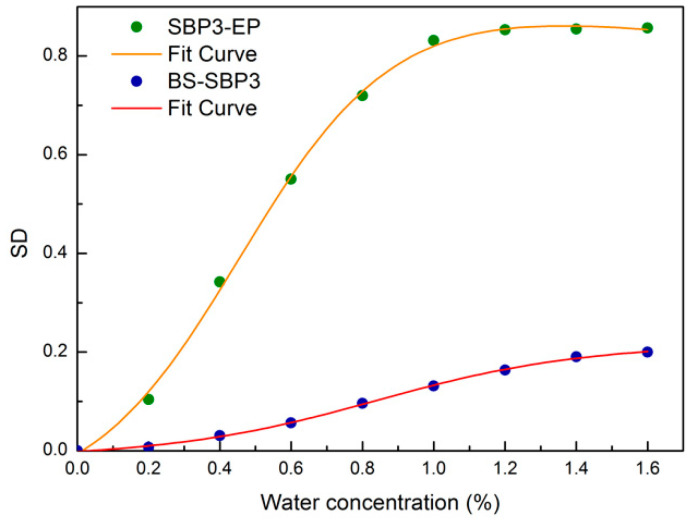
The spectral distance behavior as a function of water content concentration for the intramolecular OH-stretching bands of BS-SBP3 (blue circles) and EPS-SBP3 (green circles); fitting continuous curves for BS-SBP3 and EPS-SBP3 are shown in red and orange, respectively.

The SD behavior as a function of water content for the intramolecular OH-stretching bands of both BS-SBP3 and EPS-SBP3 is reported in Figure 7. Blue circles and green circles represent the value of SD evaluated by applying Equation (1) using as reference the anhydrous BS-SBP3 spectrum and the anhydrous SBP3-EP spectrum, respectively. As it can be seen, the evaluated SD values arrange themselves along a sigmoid curve for both the investigated systems. To extract quantitative parameters, we have fitted the experimental data utilizing Equation (2).

The fitting curve passing through the experimental data allows the extraction of the following parameters: (i) the sigmoid amplitude, (ii) the sigmoid steepness, and (iii) the inflection point. In particular, the amplitude and the steepness of the sigmoid describe the transition of water from a bound state to a free state. According to this analysis, the inflection concentration value, i.e., the sigmoid inflection point, occurs at a concentration value of 80% for BS-SBP3 and a concentration value of 48% for EPS-SBP3. This result suggests that the BS-SBP3 begins to release water molecules when 80% water is added, whereas for the EPS-SBP3 sample this occurs at a concentration of 48%.

### 3.7. Cross-Correlation Wavelet Analysis

**Figure 8:**
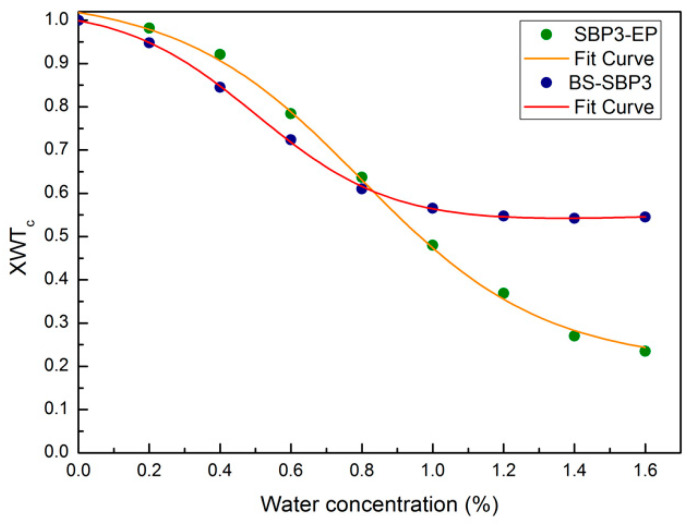
XWTC behavior as a function of water content concentration for the intramolecular OH-stretching bands for BS-SBP3 (blue circles) and EPS-SBP3 (green circles); fitting curves for BS-SBP3 and EPS-SBP3 are shown in red and orange, respectively.

To obtain different evidence for the observed behavior, an innovative approach based on the evaluation of the XWTCs has been adopted. Figure 8 shows a comparison between the values of XWTCs calculated using Equation (3) for the two investigated systems. Green circles represent the wavelet cross-correlation coefficients calculated for each couple of BS-SBP3, starting from the anhydrous spectrum BS-SBP3 and the other hydrated spectra, in the intramolecular OH-stretching region. Since the obtained data follow a descending sigmoid behavior, the model fit Equation (2) has also been applied in this case to obtain quantitative information. For the BS-SBP3, the obtained fitting curve is in orange. The same procedure has been adopted for EPS-SBP3; blue circles represent the wavelet cross-correlation coefficients, and the red curve is the fitting obtained by Equation (2).

The wavelet cross-correlation coefficient inflection point for BS-SBP3 is localized at 80%, while that for EPS-SBP3 is localized at 49%. These results agree with the results obtained by the above SD analysis and confirm the changes in the interaction between water and BS-SBP3 or EPS-SBP3.

## 4. Discussion

The shallow hydrothermal system of Panarea Island (Italy) is considered a source of microorganisms able to produce novel biopolymers [27,28,29,30,31,32,33,34]. The BSs and EPSs represent attractive biopolymers in several fields, such as bioremediation, pharmaceutics, agriculture, and cosmetics, because they are usually less toxic, more environmentally friendly, and more eco-sustainable than their industrial counterparts [15].

In this study, the polyextremophilic strain of *B. horneckiae* SBP3, previously described for its resistance to environmental and laboratory stresses [61,62], was used to produce two different polymers in two different media. It is well known that bacilli can produce a variety of exoproducts depending on the culture conditions and the extraction methods used.

The different values of ST and E_24_ of each CFS-SBP3 (Table 1) indicate that the strain produced different polymers. Previously, the strain SBP3 has been reported as an EPS producer [27] when cultivated in SWY+SAC. The lowered ST and the high kerosene-emulsifying activity (E_24_ = 62%) of CFS-MB+SAC from *B. horneckiae* SBP3 suggested the presence of BS.

The ATR-FTIR analysis showed that the structure of BS-SBP3 was attributed to a lipopeptide structure similar to surfactin, as indicated by the peaks around 1650 cm^−1^ and 2900 cm^−1^ and recently described by Zammuto et al. [35].

This type of molecule has been reported as the most potent biosurfactant produced by *Bacillus* sp. strains described for the first time by Arima et al. [63] and investigated as additives for cosmetic preparation [64], agriculture [65], and environmental applications [66]. Due to their hybrid structure and intermediate size compared to small surfactant molecules and high-molecular-weight proteins, the lipopeptide molecules diffuse and orient rapidly at the water–air and water–oil interfaces, reducing the interfacial tension [66]. Since the interfacial tension of water and air is related to the evaporation process, the lipopeptides should be considered biopolymers able to interact with water and candidates for novel wetting agents. The ability to reduce interfacial tension was also shown in this study by the contact angle experiment where the crude BS-SBP3 was able to reduce the contact angle between the hydrophobic surfaces and water from 60.2° to 29.1° at the final concentration of 10 mg/mL. This property, together with the uptake moisture that reaches 250% *w*/*w*, makes this polymer suitable as a biocompatible treatment for amending and improving the quality and wettability of soils.

The ATR-FTIR analysis of the EPS-SBP3 reveals that its spectrum possesses characteristic peaks in the spectral ranges 970–920 cm^−1^ and 1200–950 cm^−1^, which are the fingerprint regions of exopolysaccharides [36,37]. The EPSs from marine bacteria are usually characterized by different chemical compositions with many reactive groups and a great range of molecular weight conferring their diverse structures and properties. EPSs from thermophiles have been reported as biodegradable, non-toxic, safe, and environment- and human-compatible [32,33]. Furthermore, they could be used in many industrial applications such as gelling, thickeners, emulsifiers, stabilizers, and water-binding [29]. The natural conditions of extreme habitats induce the thermophiles to produce EPSs with exciting properties, including the thermostability reported for *G. thermodenitrificans* B3-72 (240 °C) [30] and *Bacillus licheniformis* T14 (240 °C) [29], that could improve their market values.

The EPS-SBP3 was shown to be able to absorb a higher quantity of water at 300% *w*/*w* but was not able to significantly modify the surface properties, as shown by the contact angle assay.

The hygroscopic properties (absorbing and retaining water) of exopolymers could be responsible for their importance in different applications ranging from agriculture to food and cosmetic industries. EPSs rich in uronic acids from thermophilic *Aeribacillus pallidus* 418 possessed hydrating properties and were considered suitable for cosmetic application; however, no study on hydrating states has been reported for these EPSs [67]. EPSs from *Streptococcus thermophilus*, *Lactobacillus delbrueckii* subsp. *bulgaricus*, and *L. casei* play a role in the food industry as stabilizers able to bind water and reduce water flow in the matrix space; for these EPSs, only gravimetrical analyses have been performed to determine their hygroscopic properties [68,69].

At the molecular level, the moisture uptake mechanisms allow the use of these biopolymers in traditional or innovative areas such as wound dressing materials or drug delivery [45]. Although the water sorption phenomenon has been characterized qualitatively and quantitatively by ATR-FTIR spectroscopy at the molecular level for cellulose [70,71], poly(vinyl alcohol), and poly(ethylene) [72], to our knowledge, no studies have been performed on bacterial exopolysaccharides or lipopeptides.

For this purpose, the intramolecular OH-stretching mode in the spectral range of 2700–3900 cm^−1^ was evaluated by ATR-FTIR at different hydrating states to determine the ability of BS-SBP3 and EPS-SBP3 to retain water

In our study, the BS-SBP3 and EPS-SBP3 showed two different behaviors when progressively hydrated. The OH-stretching band of the BS-SBP3 spectra showed two distinct bands at fixed frequencies, but with different intensities, depending on the water content, it showed a shift to higher frequencies. The OH-stretching band at 3400 cm^−1^ became more and more intense when the water content reached 80% *w*/*w*, indicating that at this hydration state, there are two populations of water molecules representing the first and the second states of hydration and that OH groups of water molecules were employed in the other chemical groups of BS-SBP3. The absorbed water in a hydrophilic polymer develops two types of hydrogen bonds; one corresponds to water molecules directly attached to the active site of the polymer to form the first hydration layer, and the other corresponds to water molecules in the second hydration layer. The latter is present in the polymer (even at low water contents). The second hydration layer can be formed on specific sites before all polar sites are saturated with water molecules [66].

The SD and XWTC analysis of BS-SBP3 suggest that the lipopeptide binds the water molecules until 80% *w*/*w*, and over this quantity (inflection point), the water molecules are released by the biopolymer. We could infer that the hydrophobic part of BS-SBP3 repulses the water molecules destabilizing the OH-bonds, whereas the hydrophilic parts bond the water to the specific polar sites.

On the contrary, the EPS-SBP3 did not show an evident shift in the frequency of the OH peak (Figure 4b). Nevertheless, the sigmoid curve of EPS-SBP3 obtained from the spectral distance and cross-correlation analysis showed a higher amplitude with the inflection point at 48% water. The analysis indicates that water molecules rapidly saturated the chemical groups of EPS-SBP3, and EPS-SBP3 was able to release the bound water molecules effortlessly.

Although the capability of BSs produced from bacilli to absorb humidity, determined using the gravimetric method, and improve soil water retention up to 314.3% and 607.7% has been previously reported [44], no information on molecular changes during water addition has been reported for those biopolymers described. Many EPSs from bacilli, such as *Paenibacillus macerans* TKU029 [73], were also reported for their potential to increase skin hydration. Water vapor permeability, water uptake rate, and water retention rate are all characteristics required to produce novel biomaterials functional as wound dressings because they should be able to absorb wound skin exudates and maintain moisture [74]. More details on molecular interaction have been reported for the hydration of lipid layers (phospholipids) and can be attributed to conformational changes of the headgroups upon hydration [75].

This study’s findings indicate that due to its ability to absorb water and modify the surface properties, the lipopeptide BS-SBP3 could be used to counter water scarcity in arid soils affected by water repellency; differently, the EPS-SBP3, due to its greater ability to absorb water, could be applied to preserve humidity in different fields such as cosmetic applications.

## 5. Conclusions

The hydrating capabilities of biopolymers play a fundamental role in industrial processes in terms of increasing the availability of water and counteracting the drying and water repellency of soils. For this purpose, the hydrating capabilities of BS-SBP3 and EPS-SBP3, produced by the marine polyextremophile *Bacillus horneckiae* SBP3 (DSM 103063), were studied using three methods: (1) wettability properties, (2) moisture absorption, and (3) hydration states.

The larger decrease in the contact angle of water on a hydrophobic surface in the presence of BS-SBP3 from 81.7° to 51.3° indicates that BS-SBP3 is capable of causing an increase in the wettability of hydrophobic surfaces, whereas the EPS-SBP3 was capable of absorbing a large quantity of water (3 times its weight) from air humidity, as revealed by moisture uptake assay, indicating its potential use as a wetting agent. The hydrating states were investigated by attenuated total reflectance Fourier-transform infrared (ATR-FTIR) spectroscopy at hydrating states ranging from 0% to 160% (*w*/*w*) water content. Spectra at different water contents were analyzed by spectral distance and wavelet cross-correlation analysis. These analyses revealed that the intramolecular OH stretching had been deconvoluted into two different contributions in the BS-SBP3, indicating an interaction between the lipopeptide and water molecules. In contrast, the OH-stretching band showed a shift in the spectrum of EPS-SBP3. At different hydrating states, the intensity of the two contributions of OH stretching, attributed to the “open” and “closed” states of water, changed in intensity over the spectra of BS-SBP3. The EPS-SBP3 spectra showed a progressive shift in the position of the whole band of OH stretching. The spectral distance and cross-correlation analyses indicate two different water behaviors of the two biopolymers, suggesting that the BS-SBP3 or EPS-SBP3 linked the water molecules differently to 80% and 48%, respectively. Due to the different hydration capabilities revealed in this study, the two biopolymers could compete with industrially manufactured additives in various fields, such as agriculture, cosmeceutical, and food industries that require moisturizing or wetting agents.

## Figures and Tables

**Table 1:** Surface tension of cell-free supernatant (CFS) from SBP3 strain, grown in seawater plus yeast extract and 3% saccharose (SWY+SAC), or marine broth plus 3% saccharose (MB+SAC); * significantly different (*p* < 0.05); ** *p* < 0.01.

Time	Surface Tension (mN m^−1^)				Emulsion Activity (E_24_)				
	CFS-SWY+SAC	CFS-MB+SAC	MB+SAC	SWY+SAC	CFS-SWY+SAC	CFS-MB+SAC	TritonX-100	SWY+SAC	MB+SAC
24	63 ± 1.2	53 ± 1.1 *	68.3 ± 1.2	67 ± 1.4	30	58	73	0	0
48	49 ± 0.6 *	39 ± 1.1 **	68.3 ± 1.6	67± 1.4	28	61	73	0	0

**Table 2:** Peak wavenumbers (cm^−1^) assigned according to Caccamo et al. [36,37], Ramani et al. [58], and Zhang et al. [59].

Wavenumber (cm^−1^)	Assignment
3700–3000	-OH stretching
2980–2920	stretching and bending vibrations of –CH
2061–1665	C-CH and O-CH stretch
1245	-CO stretch
1106	C-C -CO stretch
1060	C-O-C bend
